# Supplementary material for: Fructooligosaccharides benefits on glucose homeostasis upon high-fat diet feeding require type 2 conventional dendritic cells
Source: Nat Commun. 2024 Jun 26;15:5413. doi: 10.1038/s41467-024-49820-x (PMC11208547; doi:10.1038/s41467-024-49820-x)
Supplement: Supplementary file 1 — Supplementary Information [file 41467_2024_49820_MOESM1_ESM.pdf]

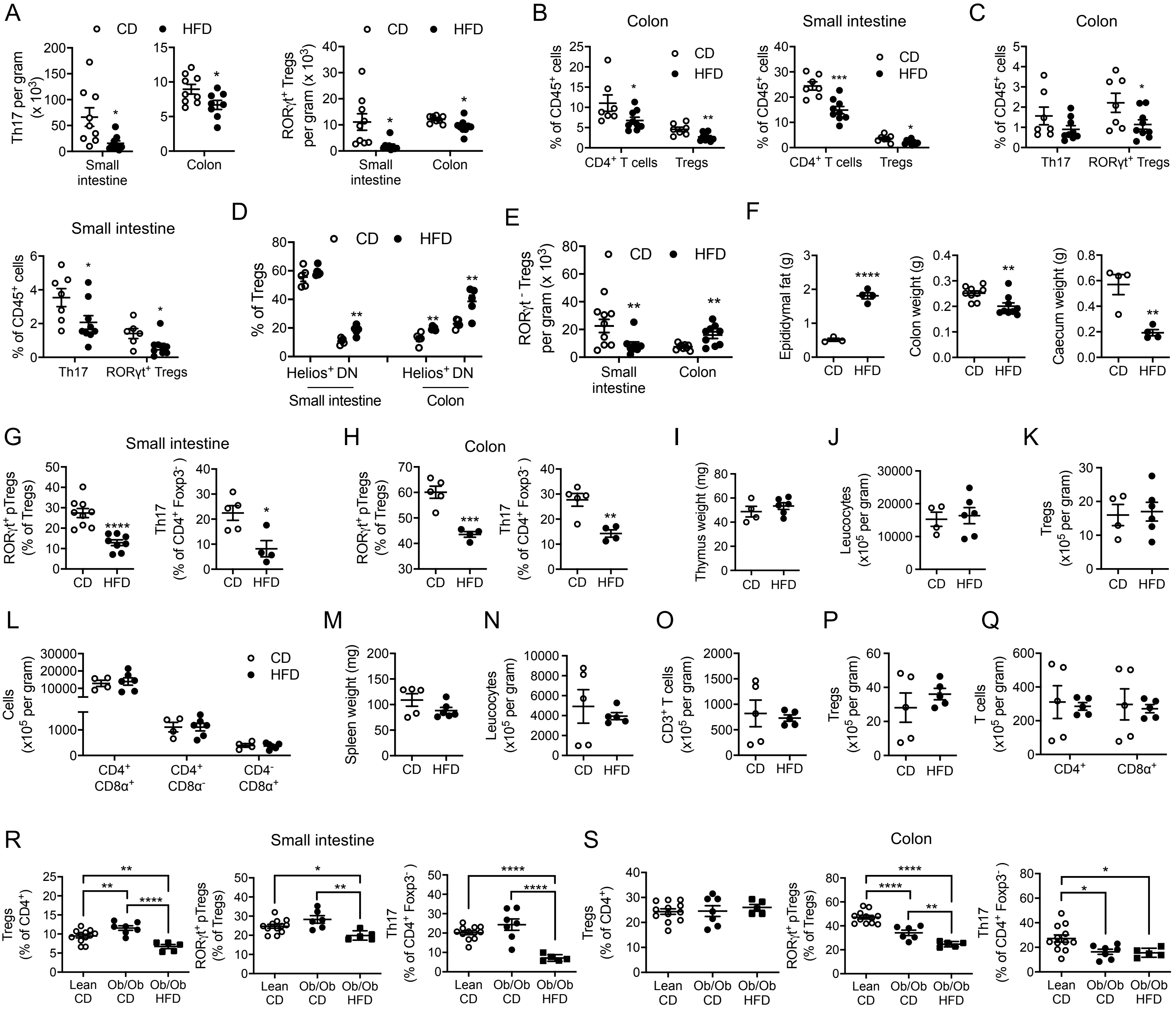

A
CD HFD
Th17 per gram (x 10^3)
Small intestine
Colon
RORγt+ Tregs per gram (x 10^3)
B
Colon
Small intestine
% of CD45+ cells
CD4+ T cells
Tregs
C
Th17
RORγt+ Tregs
D
% of Tregs
Helios+ DN
E
RORγt- Tregs per gram (x 10^3)
F
Epididymal fat (g)
Colon weight (g)
Caecum weight (g)
G
RORγt+ pTregs (% of Tregs)
Th17 (% of CD4+ Foxp3-)
H
I
Thymus weight (mg)
J
Leucocytes (x10^5 per gram)
K
Tregs (x10^5 per gram)
L
Cells (x10^5 per gram)
CD4+ CD8α+
CD4+ CD8α-
CD4- CD8α+
M
Spleen weight (mg)
N
O
CD3+ T cells (x10^5 per gram)
P
Q
T cells (x10^5 per gram)
CD4+
CD8α+
R
S
Tregs (% of CD4+)
Lean CD
Ob/Ob CD
Ob/Ob HFD

Figure S1. HFD feeding decreases RORγt$^+$ pTreg and Th17 cells in the intestine without impacting thymus and spleen T cells.

(A) Th17 and RORγt$^+$ pTregs numbers in the small intestine and colon of wild-type mice fed a chow diet (CD) or a high-fat diet (HFD) for 4 weeks (n=9 mice per group). **(B)** T cells and total Tregs expressed in proportion of CD45$^+$ leucocytes in the small intestine and colon of wild-type mice fed a CD or a HFD for 4 weeks (n=7-9 mice per group). **(C)** RORγt$^+$ pTregs and Th17 cells expressed in proportion of CD45$^+$ leucocytes in the small intestine and colon of wild-type mice fed a CD or a HFD for 4 weeks (n=7-9 mice per group). (**D**) Helios$^+$ and RORγt$^-$ Helios$^-$ double negative (DN) subsets proportion among total Tregs in the small intestine and colon of wild-type mice fed a CD or a HFD for 4 weeks (n=5 mice per group). **(E)** RORγt$^-$ Tregs numbers in the small intestine and colon of wild-type mice fed a CD or a HFD for 4 weeks (n=9 mice per group). **(F)** Epidydimal fat mass (n=3-4 mice per group, 1 experimental group), colon weight (n=9 mice per group, 2 experimental groups) and caecum weight (n=4 mice per group, 1 experimental group) in wild-type mice fed a CD or a HFD for 14 weeks. **(G)** and **(H)** Flow cytometry analysis of RORγt$^+$ pTregs and Th17 cells in the small intestine (n=9 mice per group, 2 experimental groups) and colon (n=4-5 mice per group, 1 experimental group) of wild-type mice fed a CD or a HFD for 14 weeks. **(I)** Thymus weight of wild-type mice fed CD or a HFD for 4 weeks (n=4-5 mice per group, 1 experimental group). **(J)** CD45$^+$ leucocyte numbers in the thymus of wild-type mice fed a CD or a HFD for 4 weeks (n=4-5 mice per group, 1 experimental group). **(K)** Total Treg numbers in the thymus of wild-type mice fed a CD or a HFD for 4 weeks (n=4-5 mice per group, 1 experimental group). **(L)** T cell subsets numbers in the thymus of mice a CD or a HFD for 4 weeks (n=4-5 mice per group, 1 experimental group). **(M)** Spleen weight of wild-type mice fed a CD or a HFD for 4 weeks (n=5-6 mice per group, 1 experimental group). **(N)** CD45$^+$ leucocyte numbers in the spleen of wild-type mice fed a CD or a HFD for 4 weeks (n=5-6 mice per group, 1 experimental group). **(O)** T cell numbers in the spleen of wild-type mice fed a CD or a HFD for 4 weeks (n=5-6 mice per group, 1 experimental group). **(P)** Total Treg numbers in the spleen of wild-type mice fed a CD or a HFD for 4 weeks (n=5-6 mice per group, 1 experimental group). **(Q)** CD4 and CD8α T cells in the spleen of wild-type mice fed a a CD or a HFD for 4 weeks (n=5-6 mice per group, 1 experimental group). (**R**) Flow cytometry analysis of total Tregs, RORγt$^+$ pTregs and Th17 cells in the small intestine of control mice (lean) fed a chow diet (CD) and obese *Ob/Ob* mice fed a chow diet (CD) or a high-fat diet (HFD) for 4 weeks (n=5-12 mice per group, Newman-Keuls multiple comparison test). (**S**) Flow cytometry analysis of total Tregs, RORγt$^+$ pTregs and Th17 cells in the colon of control mice (lean) fed a chow diet (CD) and obese *Ob/Ob* mice fed a chow diet (CD) or a high-fat diet (HFD) for 4 weeks (n=5-12 mice per group, Newman-Keuls multiple comparison test). All data in this figure are presented as mean values +/- SEM. Panels correspond to 2 independent experimental groups unless otherwise stated in the panel legend. All statistical tests are unpaired 2-sided T tests unless otherwise stated.

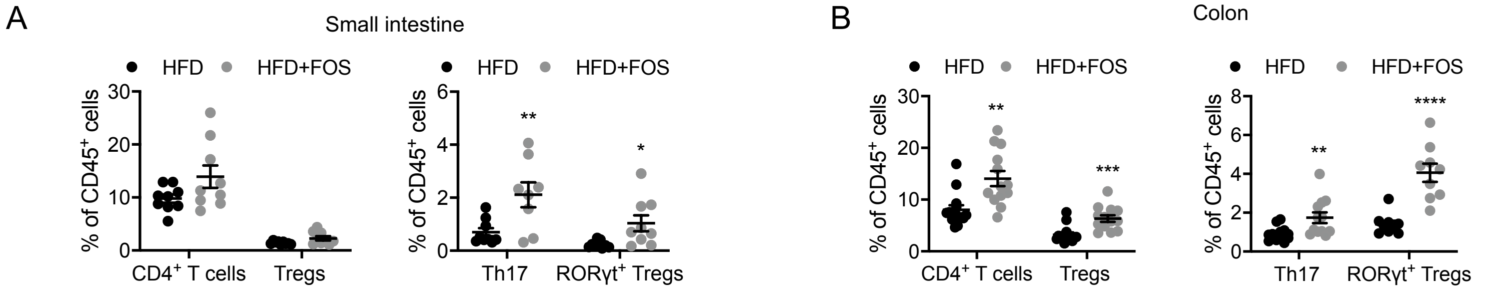

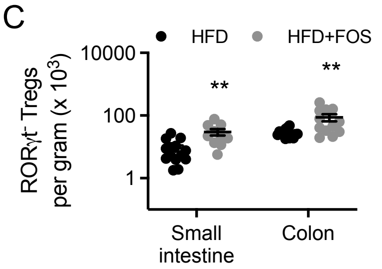

D

	Firmicutes	Proteobacteria	Bacteroidetes	Actinobacteria	Others
**CD**	42.5 ± 2.7	21.4 ± 2.5	14.6 ± 0.3	11.0 ± 0.5	10.4 ± 1.3
**HFD**	85.8 ± 3.1****	4.7 ± 1.7**	2.8 ± 0.6****	2.5 ± 0.6****	1.5 ± 0.2****
**HFD+FOS**	71.8 ± 3.3#	13.2 ± 2.9#	6 .9 ± 1.2#	4.1 ± 0.6	4.0 ± 0.6#

Multiple T tests

** P < 0.01 HFD versus CD
**** P < 0.0001 HFD versus CD
# P < 0.05 HFD+FOS versus HFD

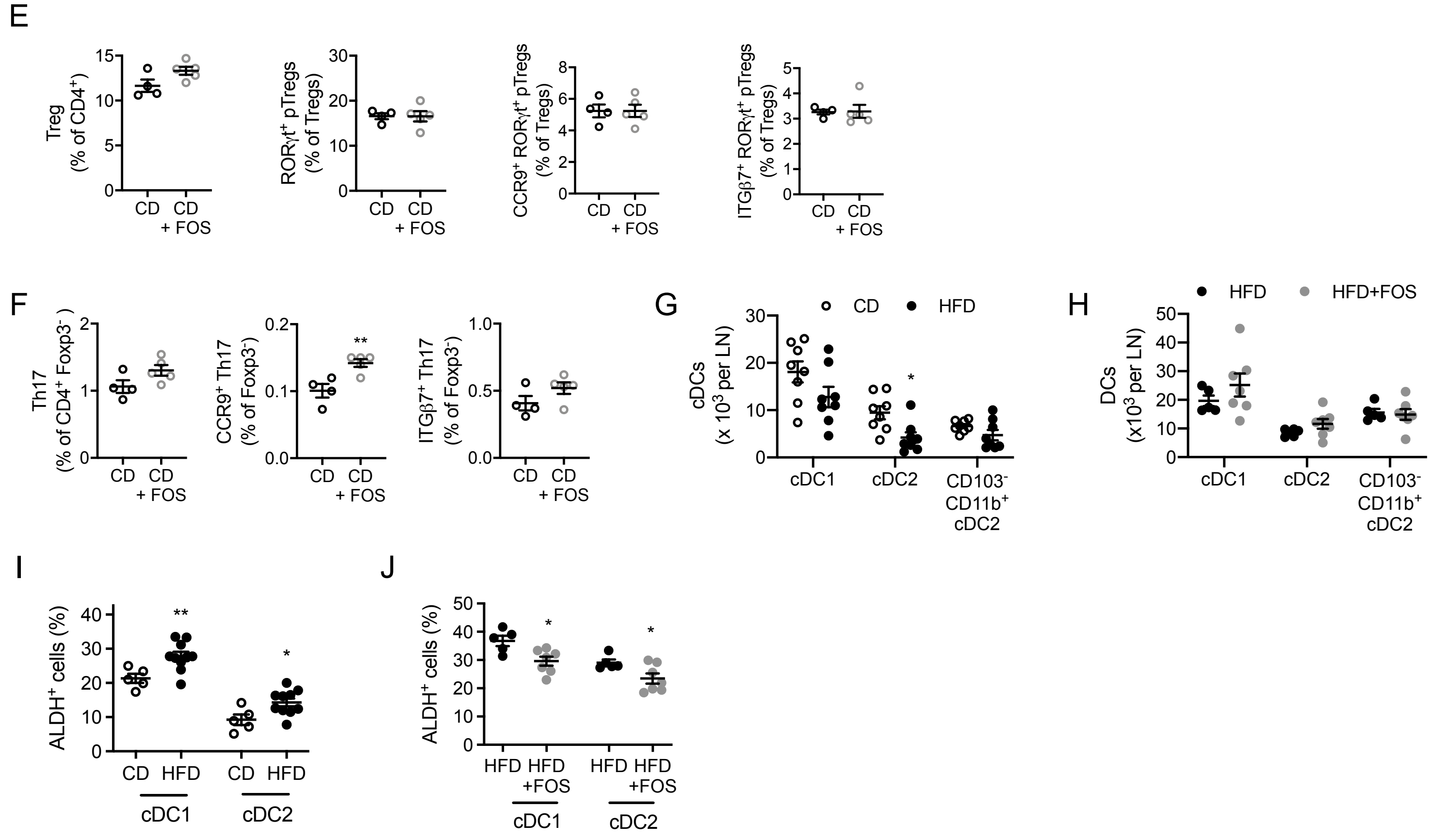

Figure S2. Impact of different diets and FOS supplementation on RORγt⁺ T cell subsets homeostasis, gut-homing imprinting as well as conventional dendritic cells counts and function.

(A and B) CD4 T cells, total Tregs, Th17 and RORγt^{+} pTregs expressed in proportion of CD45^{+} leucocytes in the small intestine (A) and colon (B) of wild-type mice fed a high-fat diet (HFD) or a high-fat diet supplemented in FOS (HFD+FOS) for 4 weeks (n=9-14 mice per group, A : 2 independent experiments, B : 2 or 3 independent experiments). **(C)** RORγt^{-} Tregs numbers in the small intestine and colon of wild-type mice fed HFD or HFD+FOS for 4 weeks (n=12-14 mice per group, 2 independent experiments). **(D)** Relative abundance of bacterial phyla in the feces of wild-type mice fed a chow diet (CD), high-fat diet (HFD) or high-fat diet supplemented in FOS (HFD+FOS) for 4 weeks (n=4 mice per group, 2 independent experiments). **(E)** Flow cytometry analysis of total Tregs, RORγt^{+} pTregs, CCR9^{+} RORγt^{+} pTregs and Itgβ7^{+} RORγt^{+} Tregs cells in the mesLNs of wild-type mice fed a chow diet (CD) or a chow diet supplemented in FOS (CD+FOS) for 4 weeks (n=4-5 mice per group, 1 experimental group). **(F)** Flow cytometry analysis of Th17 cells, CCR9^{+} Th17 cells and Itgβ7^{+} RORγt^{+} Th17 cells in the mesLNs of wild-type mice fed a CD or a CD+FOS for 4 weeks (n=4-5 mice per group, 1 experimental group). **(G)** Conventional dendritic cells (cDC) subsets (CD103^{+} CD11b^{-} cDC1, CD103^{+} CD11b^{+} cDC2 and CD103^{-} CD11b^{+} cDC2) numbers in the mesenteric lymph nodes (mesLN) of wild type mice fed a chow diet (CD) or a high-fat diet (HFD) for 4 weeks (n=8 mice per group, 2 independent experiments). **(H)** Conventional dendritic cell (cDC) numbers in the mesLNs of wild-type mice fed a high-fat diet (HFD) or a high-fat diet supplemented in FOS (HFD+FOS) for 4 weeks (n=5-7 mice per group). **(I)** ALDH-expressing (ALDH^{+}) cDC1 and CD103^{+} CD11b^{+} cDC2 in the mesLNs of wild-type mice fed a chow diet (CD) or a high-fat diet (HFD) for 4 weeks(n=5-10 mice per group, 2 independent experiments). **(J)** ALDH-expressing (ALDH^{+}) cDC1 and CD103^{+} CD11b^{+} cDC2 in the mesLNs of wild-type mice fed a high-fat diet (HFD) or a high-fat diet supplemented in FOS (HFD+FOS) for 4 weeks (n=5-7 mice per group, 2 independent experiments). All data in this figure are presented as mean values +/- SEM. All statistical tests are unpaired 2-sided T tests unless otherwise stated on the panel.

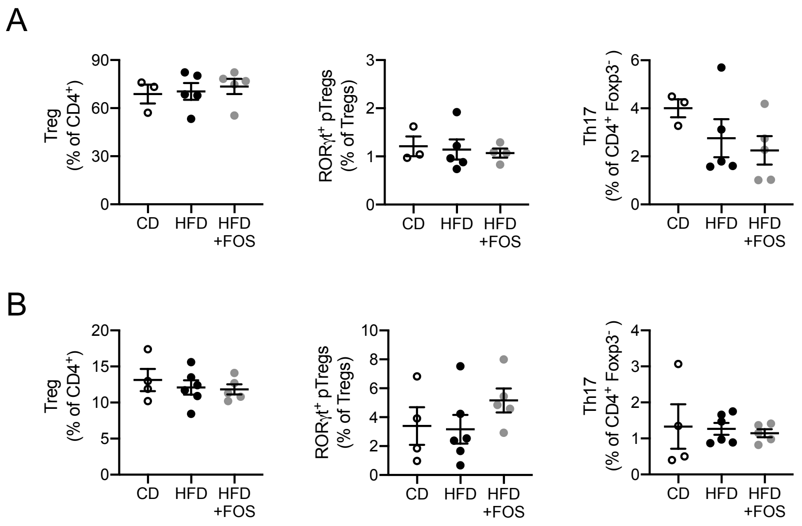

Figure S3. FOS do not impact on liver and adipose tissue RORγt^{+} pTregs and Th17 cells.

(A) Flow cytometry analysis of total Tregs, RORγt^{+} pTregs and Th17 cells in the epididymal adipose tissue of wild-type mice fed a chow diet (CD), a high-fat diet (HFD) and a high-fat diet supplemented in FOS (HFD+FOS) for 4 weeks (n=3-5 mice per group, 1 experiment). **(B)** Flow cytometry analysis of total Tregs, RORγt^{+} pTregs and Th17 cells in the liver of wild-type mice fed a chow diet (CD), a high-fat diet (HFD) or a high-fat diet supplemented in FOS (HFD+FOS) for 4 weeks (n=4-6 mice per group, 1 experiment). All data in this figure are presented as mean values +/- SEM. Statistical tests are 1-way ANOVA with Newman-Keuls multiple comparison test for all panels.

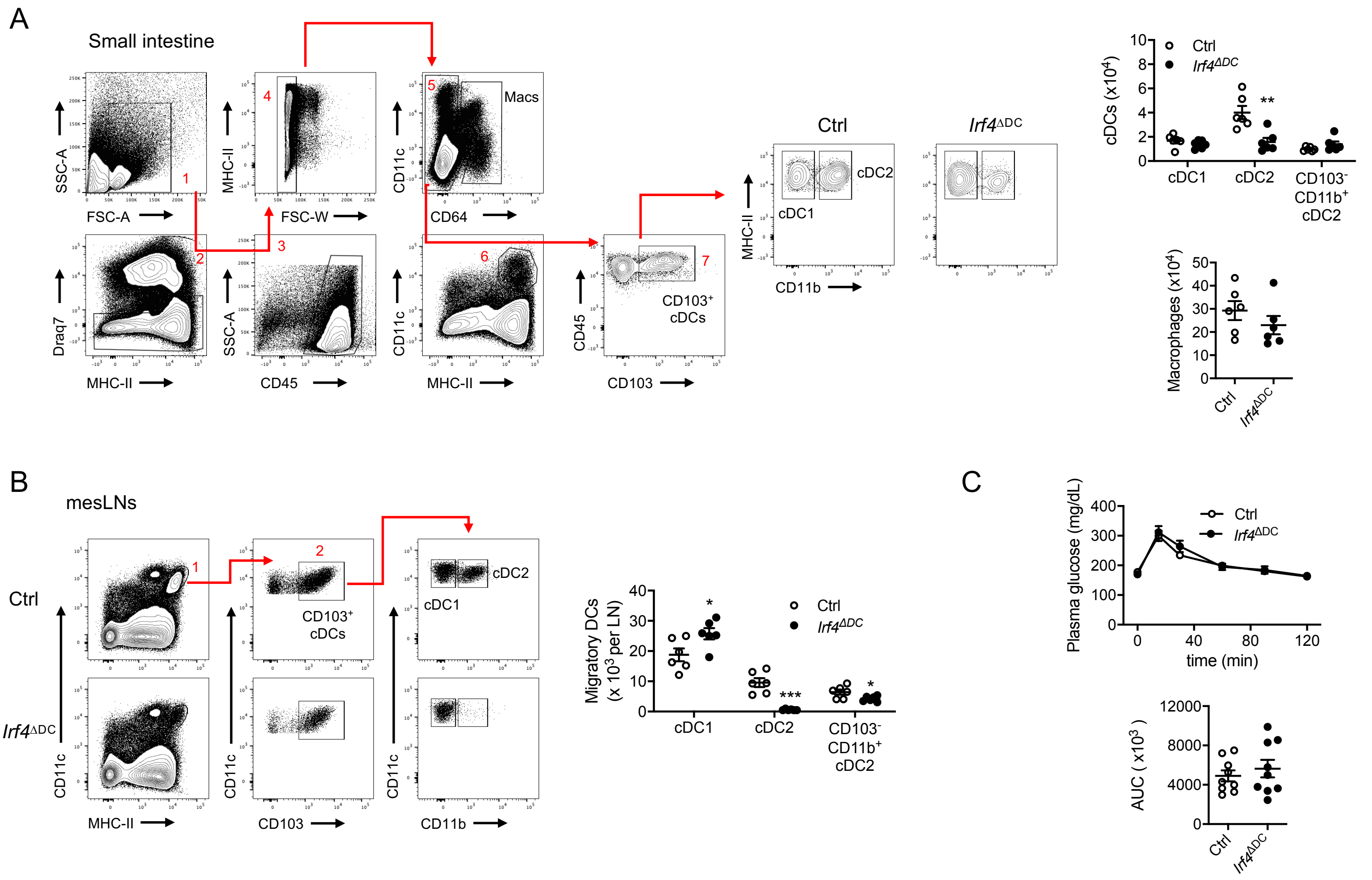

Figure S4. Dendritic cell subsets in mice invalidated for *Irf4* in CD11c-expressing cells.
(**A**) Flow cytometry analysis and quantification of dendritic cells subsets ($CD103^+$ $CD11b^-$ cDC1, $CD103^+$ $CD11b^+$ cDC2 and $CD103^-$ $CD11b^+$ cDC2) and macrophages in the small intestine of mice lacking *Irf4* in dendritic cells ($Irf4^{\Delta DC}$) and $Irf4^{flox/flox}$ controls (Ctrl) (n=6 mice per group). (**B**) Flow cytometry analysis and quantification of dendritic cells subsets ($CD103^+$ $CD11b^-$ cDC1, $CD103^+$ $CD11b^+$ cDC2 and $CD103^-$ $CD11b^+$ cDC2) in the mesenteric lymph nodes (mesLNs) of mice lacking *Irf4* in dendritic cells ($Irf4^{\Delta DC}$) and $Irf4^{flox/flox}$ controls (Ctrl) (n=6 mice per group, 2 independent experiments). (**C**) Oral glucose tolerance test and corresponding area under curve (AUC) of mice lacking *Irf4* in dendritic cells ($Irf4^{\Delta DC}$) and $Irf4^{flox/flox}$ controls (Ctrl) fed a chow diet (n=9 mice per group, 2 independent experiments). All data in this figure are presented as mean values +/- SEM. All statistical tests are unpaired 2-sided T tests.

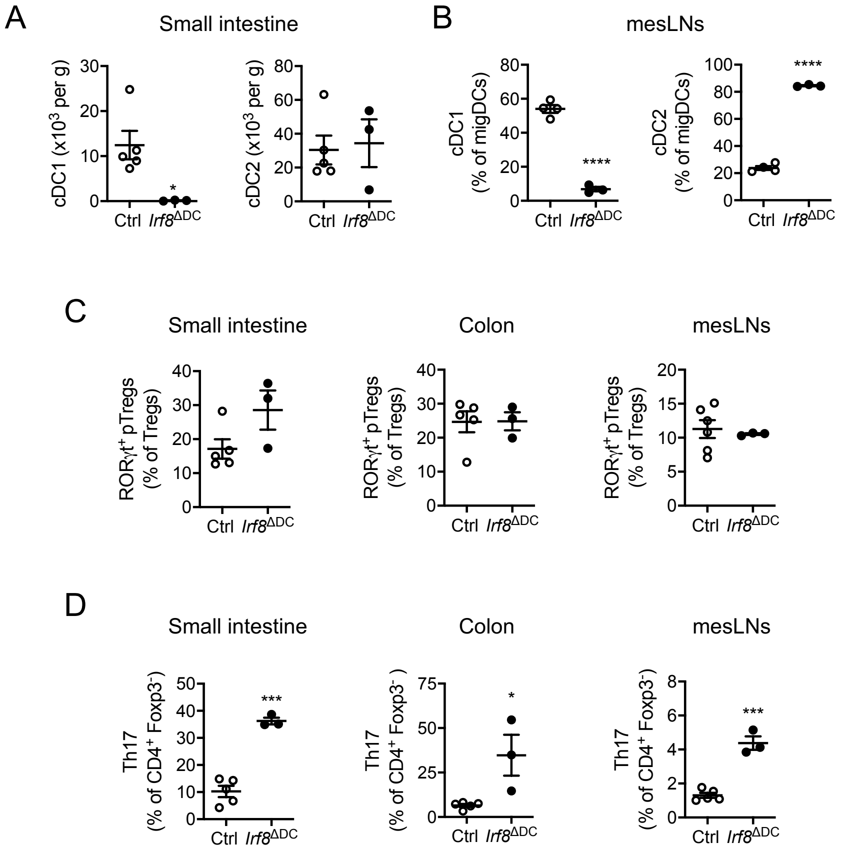

Figure S5. Analysis of $ROR\gamma t^+$ pTregs and Th17 cells in mice devoid of cDC1.
(**A** and **B**) Dendritic cells subsets ($CD103^+$ $CD11b^-$ cDC1 and $CD103^+$ $CD11b^+$ cDC2) in the small intestine (A) and mesenteric lymph nodes (mesLNs) of mice lacking *Irf8* in dendritic cells (*Irf8*$^{\Delta DC}$) and *Irf8*$^{flox/flox}$ controls (Ctrl) (n=4-5 mice per group, 1 experiment). (**C** and **D**) Flow cytometry analysis of $ROR\gamma t^+$ pTreg (C) and Th17 cells (D) in the small intestine, colon and mesenteric lymph nodes (mesLNs) of mice lacking *Irf8* in dendritic cells (*Irf8*$^{\Delta DC}$) and *Irf8*$^{flox/flox}$ controls (Ctrl) (n=3-5 mice per group, 1 experiment). All data in this figure are presented as mean values +/- SEM. All statistical tests are unpaired 2-sided T tests.

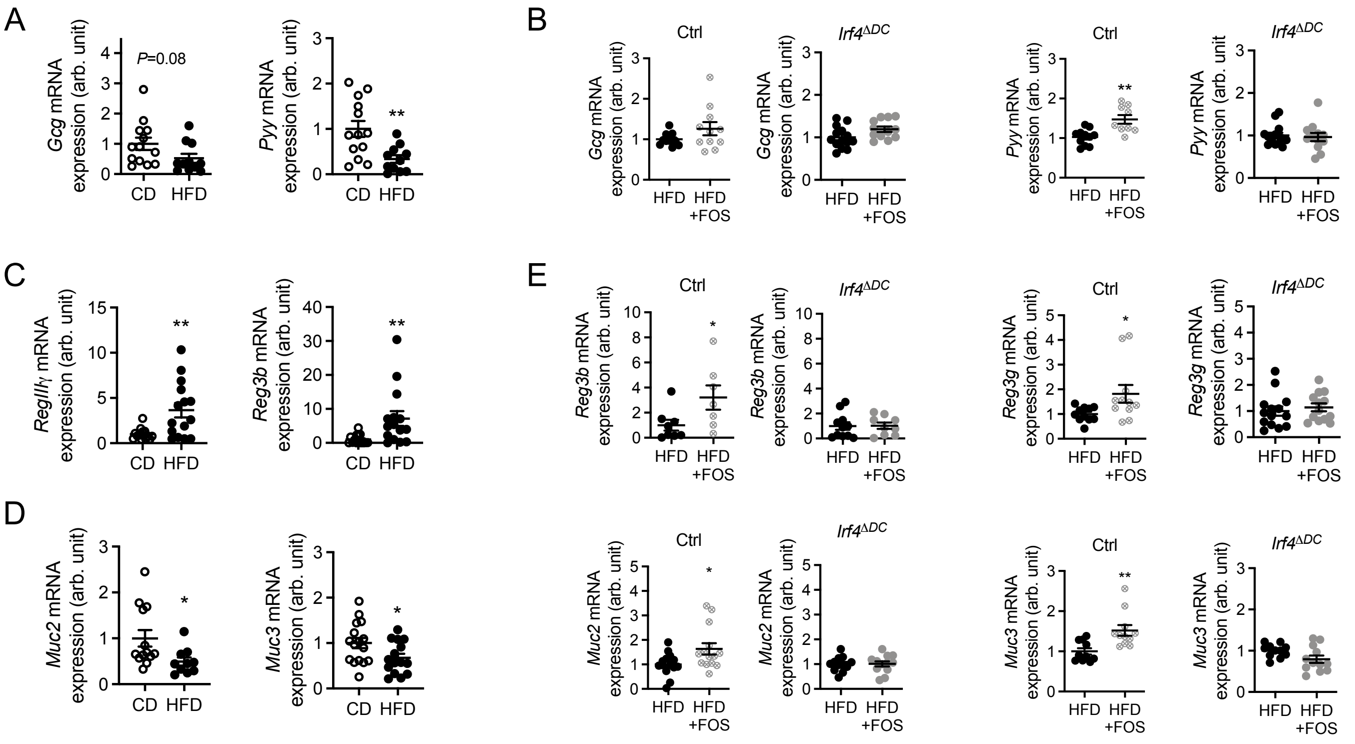

Figure S6. cDC2 mediate the regulation of gut function-related genes by FOS.
(**A**) *Gcg* and *Pyy* mRNA expression measured by RT-qPCR in the colon of wild-type mice fed a chow diet (CD) or a high-fat diet (HFD) for 4 weeks (n=11-13 mice per group). (**B**) *Gcg* and *Pyy* mRNA expression measured by RT-qPCR in the colon of *Irf4*$^{flox/flox}$ control animals (Ctrl) and *Irf4*$^{\Delta DC}$ mice fed a high-fat diet (HFD) or a high-fat diet supplemented in FOS (HFD+FOS) for 4 weeks (n=11-14 mice per group). (**C**) *Reg3g* and *Reg3b* mRNA expression measured by RT-qPCR in the colon of wild-type mice fed a chow diet (CD) or a high-fat diet (HFD) for 4 weeks (n=14-15 mice per group). (**D**) *Muc2* and *Muc3* mRNA expression measured by RT-qPCR in the colon of wild-type mice fed a chow diet (CD) or a high-fat diet (HFD) for 4 weeks (n=10-16 mice per group). (**E**) *Reg3b*, *Reg3g*, *Muc2* and *Muc3* mRNA expression measured by RT-qPCR in the colon of *Irf4*$^{flox/flox}$ control animals (Ctrl) and *Irf4*$^{\Delta DC}$ mice fed a high-fat diet (HFD) or a high-fat diet supplemented in FOS (HFD+FOS) for 4 weeks (n=7-15 mice per group, 2 independent experiments). All data in this figure are presented as mean values +/- SEM. All statistical tests are unpaired 2-sided T tests. All panels correspond to minimum 2 independent experimental groups. Arb.units: arbitrary units.
