# Supplementary material for: Fructooligosaccharides benefits on glucose homeostasis upon high-fat diet feeding require type 2 conventional dendritic cells
Source: Nat Commun. 2024 Jun 26;15:5413. doi: 10.1038/s41467-024-49820-x (PMC11208547; doi:10.1038/s41467-024-49820-x)
Supplement: Supplementary file 3 — Reporting Summary [file 41467_2024_49820_MOESM3_ESM.pdf]

nature portfolio

Corresponding author(s): E. Gautier

Last updated by author(s): Mar 8, 2024

Reporting Summary

Nature Portfolio wishes to improve the reproducibility of the work that we publish. This form provides structure for consistency and transparency in reporting. For further information on Nature Portfolio policies, see our Editorial Policies and the Editorial Policy Checklist.

Statistics

For all statistical analyses, confirm that the following items are present in the figure legend, table legend, main text, or Methods section.

n/a	Confirmed	
☐	☒	The exact sample size (*n*) for each experimental group/condition, given as a discrete number and unit of measurement
☐	☒	A statement on whether measurements were taken from distinct samples or whether the same sample was measured repeatedly
☐	☒	The statistical test(s) used AND whether they are one- or two-sided<br>*Only common tests should be described solely by name; describe more complex techniques in the Methods section.*
☐	☒	A description of all covariates tested
☐	☒	A description of any assumptions or corrections, such as tests of normality and adjustment for multiple comparisons
☐	☒	A full description of the statistical parameters including central tendency (e.g. means) or other basic estimates (e.g. regression coefficient) AND variation (e.g. standard deviation) or associated estimates of uncertainty (e.g. confidence intervals)
☐	☒	For null hypothesis testing, the test statistic (e.g. *F*, *t*, *r*) with confidence intervals, effect sizes, degrees of freedom and *P* value noted<br>*Give P values as exact values whenever suitable.*
☒	☐	For Bayesian analysis, information on the choice of priors and Markov chain Monte Carlo settings
☒	☐	For hierarchical and complex designs, identification of the appropriate level for tests and full reporting of outcomes
☒	☐	Estimates of effect sizes (e.g. Cohen's *d*, Pearson's *r*), indicating how they were calculated

Our web collection on statistics for biologists contains articles on many of the points above.

Software and code

Policy information about availability of computer code

Data collection	FACS Diva software (v8.0.2) was used for flow cytometry data collection.
Data analysis	FlowJo software (v10) was used for flow cytometry data analysis.<br>R studio software (v2023.12.1) was used to analyze microbiome sequencing data according to Alili R. et al. Genes 2021 by using the Nanopore.2.0 pipeline available online (https://git.ummisco.fr/ebelda/nanopore.v2.0) .<br>Prism software (v8) was used for statistical analysis.

For manuscripts utilizing custom algorithms or software that are central to the research but not yet described in published literature, software must be made available to editors and reviewers. We strongly encourage code deposition in a community repository (e.g. GitHub). See the Nature Portfolio guidelines for submitting code & software for further information.

Data

Policy information about availability of data

All manuscripts must include a data availability statement. This statement should provide the following information, where applicable:
- Accession codes, unique identifiers, or web links for publicly available datasets
- A description of any restrictions on data availability
- For clinical datasets or third party data, please ensure that the statement adheres to our policy

The data are availalble from the corresponding author upon reasonable request. Microbiota sequencing data were deposited on the Sequence Read Archive (SRA) (PRJNA1099178, https://www.ncbi.nlm.nih.gov/sra/PRJNA1099178).

Research involving human participants, their data, or biological material

Policy information about studies with human participants or human data. See also policy information about sex, gender (identity/presentation), and sexual orientation and race, ethnicity and racism.

Reporting on sex and gender	Not applicable
Reporting on race, ethnicity, or other socially relevant groupings	Not applicable
Population characteristics	Not applicable
Recruitment	Not applicable
Ethics oversight	Not applicable

Note that full information on the approval of the study protocol must also be provided in the manuscript.

Field-specific reporting

Please select the one below that is the best fit for your research. If you are not sure, read the appropriate sections before making your selection.

☒ Life sciences ☐ Behavioural & social sciences ☐ Ecological, evolutionary & environmental sciences

For a reference copy of the document with all sections, see nature.com/documents/nr-reporting-summary-flat.pdf

Life sciences study design

All studies must disclose on these points even when the disclosure is negative.

Sample size	Sample size calculation was not performed but was based on our previous expertise as well as previous studies (Marcelin G et al. Cell Metabolism 2017 ; Tran S et al. Immunity 2020 ; Selimkhanov J et al. Int J Obes 2017 ; Tschöp MH et al. Nat Methods 2011)
Data exclusions	No
Replication	Individual animals were studied and experiments were replicated at least two times.
Randomization	Animal cages were distributed randomly across treatment groups and animals were assigned to group based on their genetic background when necessary. Single animals couldn't be randomized when males were studied as the full cage was assigned to a particular treatment.
Blinding	Blinding was not performed because the investigator who performed the experiments was the person making the analysis and plans. However, data analysis was based on objectively measurable readouts without subjective interpretation, and samples were processed in a random fashion.

Reporting for specific materials, systems and methods

We require information from authors about some types of materials, experimental systems and methods used in many studies. Here, indicate whether each material, system or method listed is relevant to your study. If you are not sure if a list item applies to your research, read the appropriate section before selecting a response.

Materials & experimental systems

n/a	Involved in the study
	☒ Antibodies
☒	☐ Eukaryotic cell lines
☒	☐ Palaeontology and archaeology
	☒ Animals and other organisms
☒	☐ Clinical data
☒	☐ Dual use research of concern
☒	☐ Plants

Methods

n/a	Involved in the study
☒	☐ ChIP-seq
	☒ Flow cytometry
☒	☐ MRI-based neuroimaging

Antibodies

Antibodies used

CCR9 PerCP-eFluor710, clone CW-1.2, Cat#46-1991-82, eBiosciences
ITGb7 PE, clone DATK32, Cat#120606 BioLegend,
CD64 PE, clone X54-5/7.1, Cat#139304, BioLegend
CD45 BV510, clone 30-F11, Cat#103138, BioLegend
CD45 APC/Fire750, clone 30-F11, Cat#103154, BioLegend
CD11c BV605, clone N418, Cat#117334, BioLegend
MHC-II, alias I-A/I-E APC/Fire750, clone M5/114.15.2, Cat#107652, BioLegend
CD103 FITC, clone 2E7, Cat#121420, BioLegend
CD11b AlexaFluor700, clone M1/70, Cat#101222, BioLegend
RORγt AlexaFluor647, clone Q.31-378, Cat#562682, BD Biosciences
Foxp3 eFluor450, clone FJK-16s, Cat#5016373, Invitrogen
CD4 BV510, clone RM4-5, Cat#100559, BioLegend
CD4 AlexaFluor700, clone RM4-5, Cat#100536, BioLegend

Validation

CCR9 PerCP-eFluor710, clone CW-1.2 https://www.thermofisher.com/antibody/product/CD199-CCR9-Antibody-clone-eBioCW-1-2-CW-1-2-Monoclonal/46-1991-82
ITGb7 PE, clone DATK32 https://www.biolegend.com/en-gb/products/pe-anti-mouse-lpam-1-integrin-alpha4beta7-antibody-2971?GroupID=BLG15664
CD64 PE, clone X54-5/7.1 https://www.biolegend.com/de-de/products/pe-anti-mouse-cd64-fcgammari-antibody-6691?GroupID=BLG8810
CD45 BV510, clone 30-F11 https://www.biolegend.com/en-ie/products/brilliant-violet-510-anti-mouse-cd45-antibody-7995
CD45 APC/Fire750, clone 30-F11 https://www.biolegend.com/fr-lu/products/apc-fire-750-anti-mouse-cd45-antibody-13049
CD11c BV605, clone N418 https://www.biolegend.com/nl-be/products/brilliant-violet-605-anti-mouse-cd11c-antibody-7865?GroupID=BLG11937
MHC-II, alias I-A/I-E, clone M5/114.15.2 https://www.biolegend.com/nl-be/products/apc-fire-750-anti-mouse-i-a-i-e-antibody-13215?GroupID=BLG11931
CD103 FITC, clone 2E7 https://www.biolegend.com/de-at/products/fitc-anti-mouse-cd103-antibody-7053?GroupID=BLG4646
CD11b AlexaFluor700, clone M1/70 https://www.biolegend.com/nl-be/products/alexa-fluor-700-anti-mouse-human-cd11b-antibody-3388
RORγt AlexaFluor647, clone Q.31-378 https://www.bdbiosciences.com/en-us/products/reagents/flow-cytometry-reagents/research-reagents/single-color-antibodies-ruo/alexa-fluor-647-mouse-anti-mouse-ror-t.562682
Foxp3 eFluor450, clone FJK-16s https://www.fishersci.ca/shop/products/foxp3-monoclonal-antibody-fjk-16s-efluor-450-ebioscience-invitrogen/p-7091182
CD4 BV510, clone RM4-5 https://www.biolegend.com/de-at/products/brilliant-violet-510-anti-mouse-cd4-antibody-7991?GroupID=BLG4745
CD4 AlexaFluor700, clone RM4-5 https://www.biolegend.com/nl-be/products/alexa-fluor-700-anti-mouse-cd4-antibody-3386

Animals and other research organisms

Policy information about studies involving animals; ARRIVE guidelines recommended for reporting animal research, and Sex and Gender in Research

Laboratory animals

Mice were housed in individually ventilated cages at a temperature of 22°C and humidity around 50%. They were maintained under specific pathogen-free conditions on a 12-hour light and dark cycle with ad libitum access to water and diet (A04; Safe-Diets). Wild-type C57BL/6J mice were from Charles River and bred in-house. Ob/+ (B6.Cg-Lepob/J) mice were from Charles River and bred in house to generate obese Ob/Ob mice and lean littermate controls (including Ob/+ and +/+ animals). Jackson-originating Itgax-cre x Irf4flox/flox animals were bred in our facility. Itgax-cre x Irf8flox/flox were directly imported from the Tussiwand lab (Basel Institute, Switzerland). All mice were on a C57Bl6 background. Animals between 10 and 14 weeks of age were used for experiments.
All animal procedures were in accordance with the Guide for the Care and Use of Laboratory Animals published by the European Commission Directive 86/609/EEC and given authorization from the French Ministry of Research and local ethics committee (Charles Darwin, CEEA - 005).

Wild animals

No wild animals were used in the study.

Reporting on sex	To avoid hormonal bias and in accordance with most of the literature, obesity studies were performed only in male mice.
Field-collected samples	No field collected samples were used in the study.
Ethics oversight	All animal procedures were in accordance with the Guide for the Care and Use of Laboratory Animals published by the European Commission Directive 86/609/EEC and given authorization from the French Ministry of Research and local ethics committee (Charles Darwin, CEEA - 005)

Note that full information on the approval of the study protocol must also be provided in the manuscript.

Plants

Seed stocks	Not applicable
Novel plant genotypes	Not applicable
Authentication	Not applicable

Flow Cytometry

Plots

Confirm that:

- [x] The axis labels state the marker and fluorochrome used (e.g. CD4-FITC).
- [x] The axis scales are clearly visible. Include numbers along axes only for bottom left plot of group (a 'group' is an analysis of identical markers).
- [x] All plots are contour plots with outliers or pseudocolor plots.
- [x] A numerical value for number of cells or percentage (with statistics) is provided.

Methodology

Sample preparation	*Describe the sample preparation, detailing the biological source of the cells and any tissue processing steps used.*
Instrument	FACS Diva was used for data collection.
Software	FACS Diva software was used for data collection.<br>FlowJo software was used for data analysis.
Cell population abundance	From 0.2 to 1 x 10^6 total cells were collected to obtain sufficient numbers of the target cell population.
Gating strategy	Gating strategies were described in the text as well as in supplemental figures.

- [x] Tick this box to confirm that a figure exemplifying the gating strategy is provided in the Supplementary Information.
